# Supplementary material for: Enhanced T-Cell Priming and Improved Anti-Tumor Immunity through Lymphatic Delivery of Checkpoint Blockade Immunotherapy
Source: Cancers (Basel). 2022 Apr 4;14(7):1823. doi: 10.3390/cancers14071823 (PMC8997812; doi:10.3390/cancers14071823)
Supplement: Supplementary file 1 [file cancers-14-01823-s001.zip › cancers-1628057-supp-final/cancers-1628057-supp-final.pdf]

Supplementary Materials

Enhanced T-Cell Priming and improved Anti-Tumor Immunity through Lymphatic Delivery of Checkpoint Blockade Immunotherapy

Carolina Mantilla-Rojas, Fred C. Velasquez, Janelle Morton, Leticia C. Clemente, Edwin R. Parra, Carlos Torres-Cabala and Eva M. Sevick-Muraca

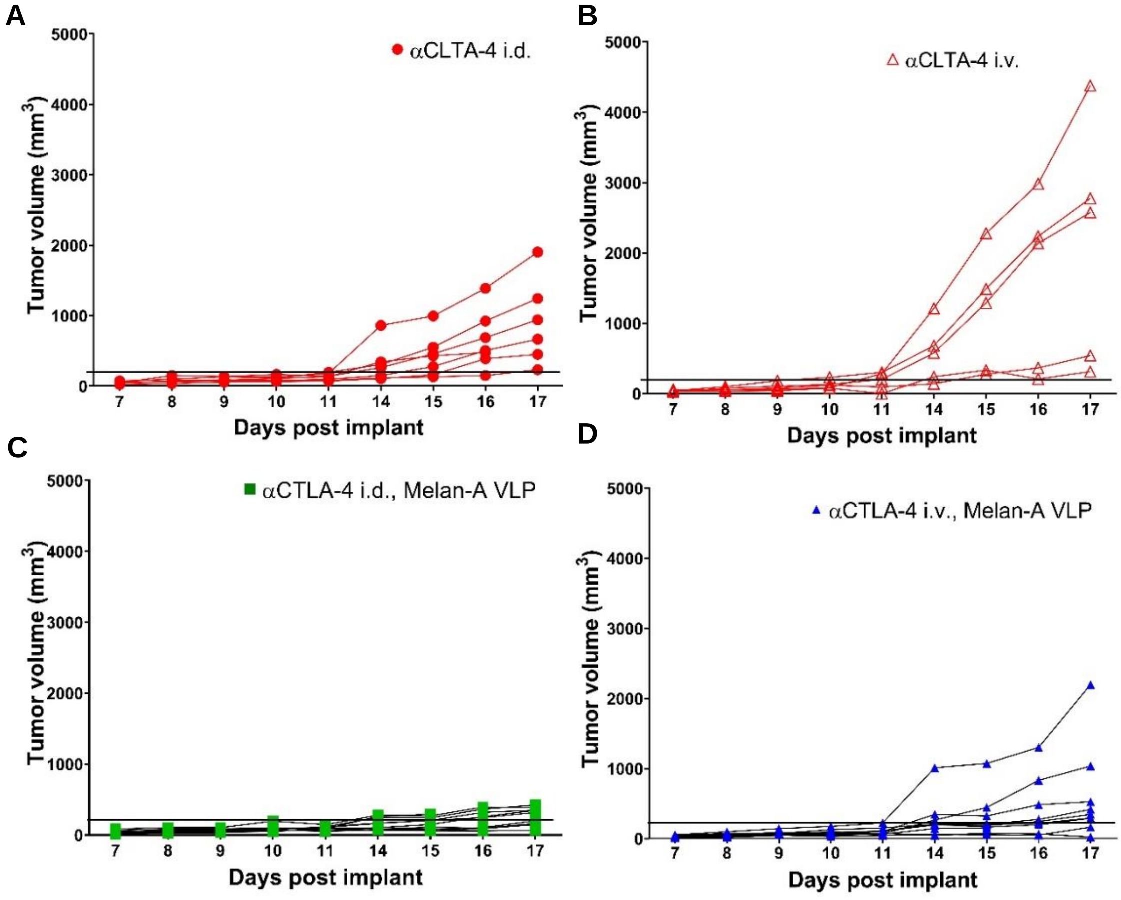

Figure S1. Primary tumor growth in animals dosed with αCTLA-4 (**A**) intradermal (i.d.) administration; (**B**) intravenous (i.v.) administration; (**C**) i.d. with Melan-A VLP; and (**D**) i.v. with Melan-A VLP.

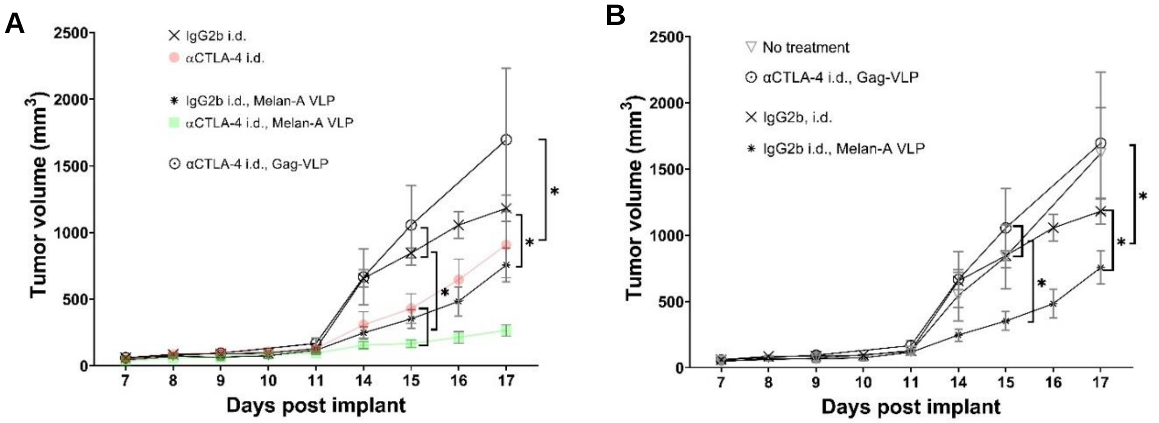

Figure S2. (**A**,**B**) Lymphatic delivery of Melan-A VLP alone or in combination with αCTLA-4 administered intradermal (i.d.), significantly reduces primary tumor growth. No-treatment (▽, grey, $n = 5$); IgG2b i.d. (X, $n = 4$); IgG2b i.d., Melan-A VLP (✱, $n = 4$); αCTLA-4 i.d (●, red, $n = 7$); αCTLA-4 i.d., VLP i.d. (■, green, $n = 10$); and αCTLA-4 i.d., Gag-VLP (○, $n = 10$). * denotes statistical significance by unpaired, two-tailed Student's T test ($p < 0.05$). Error bars indicate SEM.

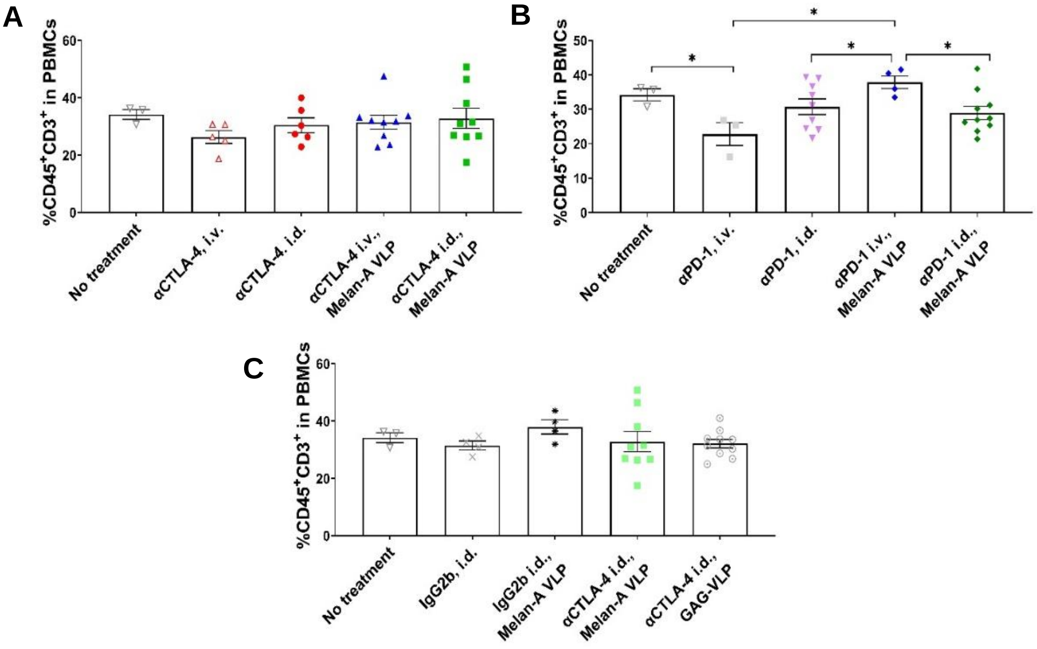

Figure S3. %CD45$^+$ CD3$^+$ cells in PBMCs. (**A**) Lymphatic delivery of αCTLA-4 monotherapy and in combination with Melan-A VLP does not change %CD45$^+$CD3$^+$ T cells in PBMCs. No treatment (▽, grey, $n = 3$); αCTLA-4 administered intravenously (i.v.) (△, red, $n = 5$); αCTLA-4 administered intradermally (i.d.)(●, red, $n = 6$); αCTLA-4 i.v., Melan-A VLP i.d (▲, blue, $n = 9$); αCTLA-4 i.d., and Melan-A VLP i.d. (■, green, $n = 9$). (**B**) Lymphatic delivery of αPD-1 changes the %CD45$^+$CD3$^+$ in PBMCs. No-treatment (▽, grey, $n = 3$); αPD-1 i.v (■, grey, $n = 3$); αPD-1 i.d (▼, pink, $n = 9$); αPD-1 i.v., Melan-A VLP i.d (◆, blue, $n = 4$); and αPD-1 i.d., Melan-A VLP i.d. (◆, green, $n = 10$). (**C**) Lymphatic delivery of αCTLA-4 monotherapy with Melan-A VLP does not change the %CD45$^+$CD3$^+$ in PBMCs. No-treatment (▽, grey $n = 3$); IgG2b i.d. (X, $n = 4$); IgG2b i.d., Melan-A VLP(✱, $n = 4$); αCTLA-4 i.d., VLP i.d. (■, green, $n = 9$); and αCTLA-4 i.d., gag VLP(○, $n = 9$). * denotes statistical significance by unpaired, two-tailed Student's T test ($p < 0.05$). Error bars indicate SEM.

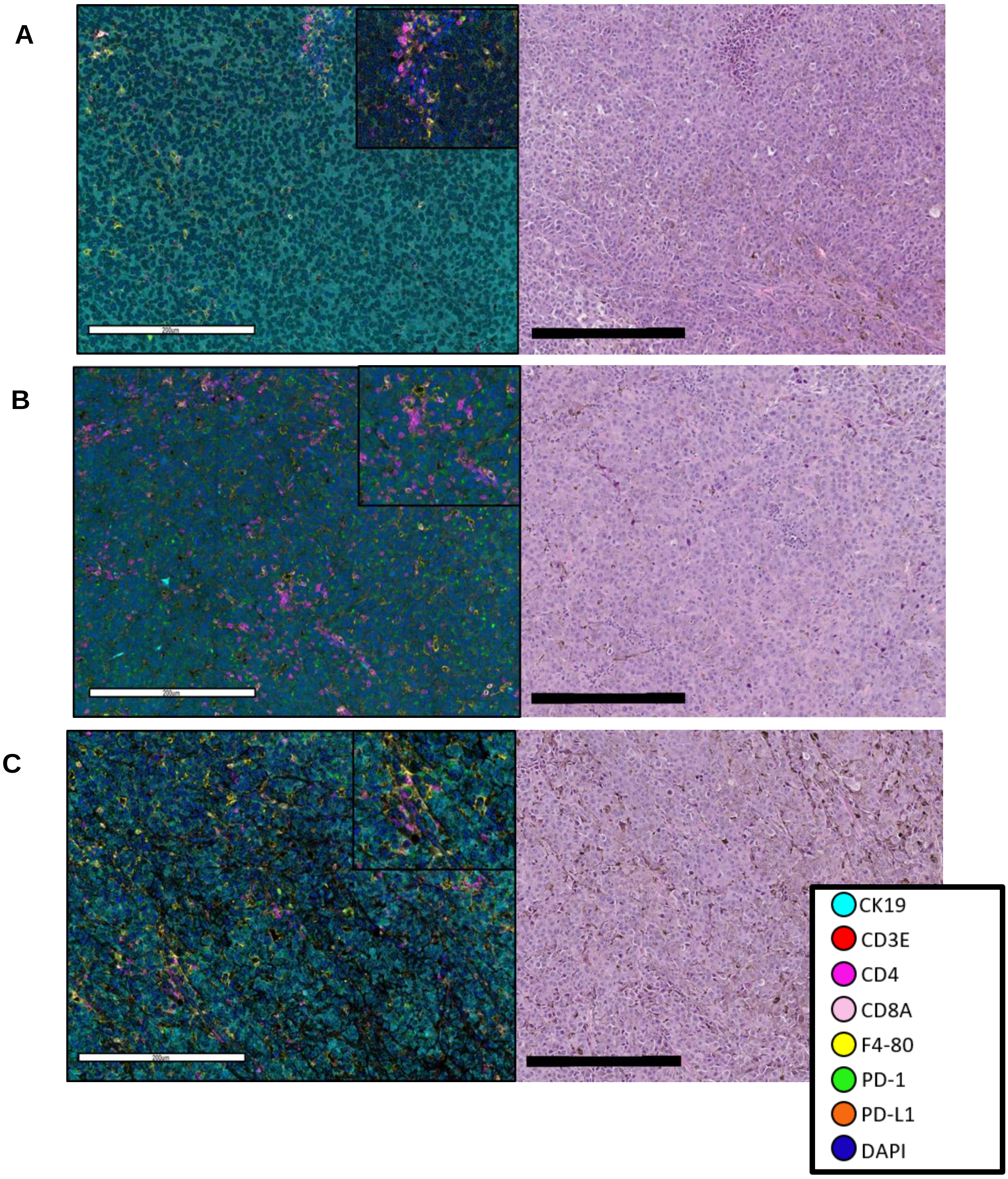

Figure S4. Example multiplex immunofluorescence (mIF, left column) and H&E (right column) of tumor tissue harvested at 17 days post implant in animals treated with (**A**) αCTLA-4 administered intradermally (i.d.) with gag VLP; (**B**) αCTLA-4 i.d., with Melan-A VLP; and (**C**) αCTLA-4 administered intravenously (i.v.) with Melan-A VLP. Scale bars on mIF (white) and H&E (black) represent 200 µm.

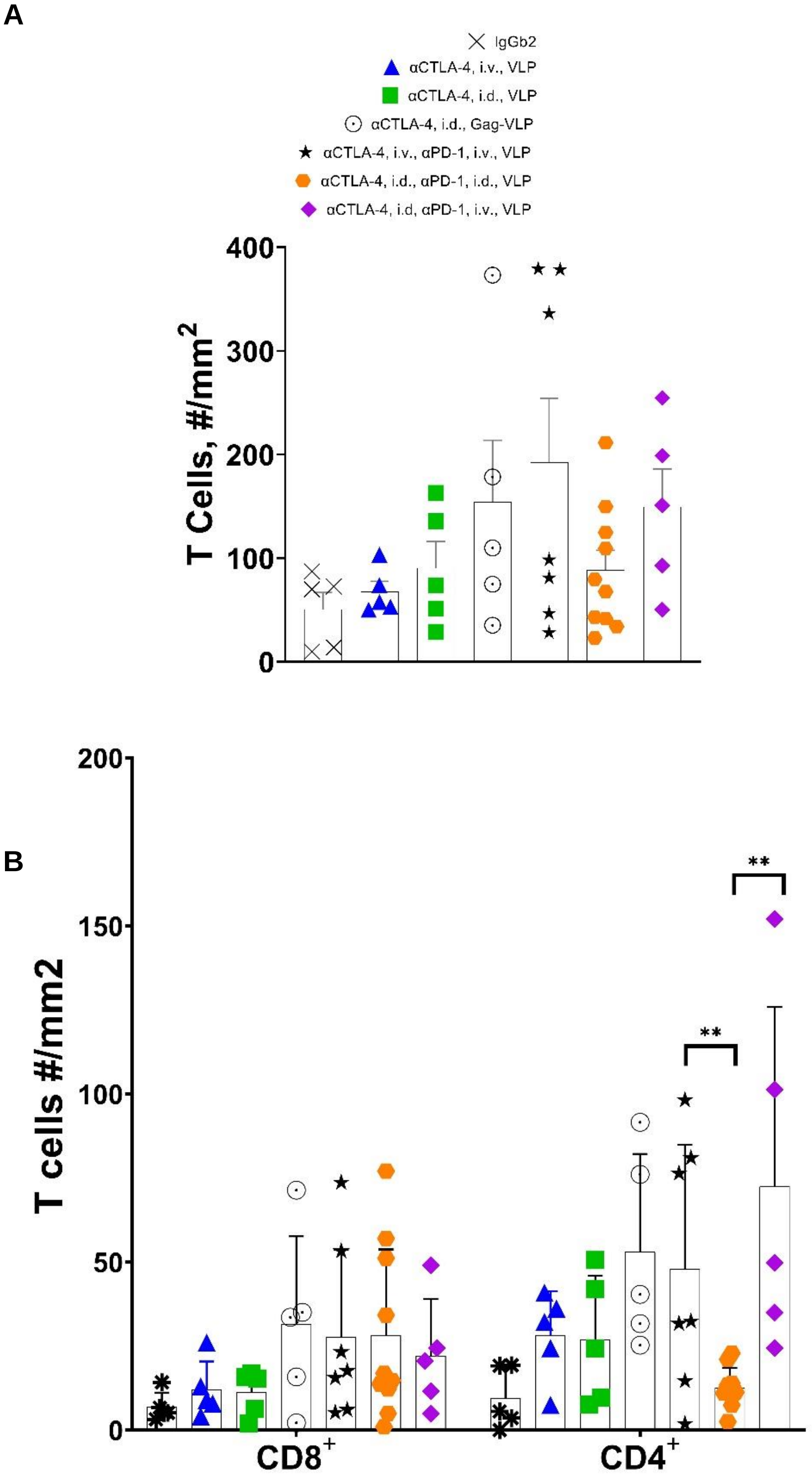

Figure S5. (**A**) $CD45^+CD3E^+$ T cells (#/mm^2) at 17 days post implant (d.p.i.) (**B**) CD4+ and CD8+ T cells (#/mm2) in the tumor microenvrionment at 17 d.p.i. Data from animals dosed with IgG2b, administered intradermally (i.d.) (X, n = 5); αCTLA-4 administered intravenously (i.v.), Melan-A VLP (▲, blue, n = 5); αCTLA-4 i.d., Melan-A VLP (■, green n = 5); αCTLA-4 i.d., Gag-VLP (○, n = 5); αCTLA-4 i.v., αPD-1 i.v., Melan-A VLP (★, black, n = 7); αCTLA-4 i.d., αPD-1 i.d., Melan-A VLP (⬣, orange n = 10); and αCTLA-4 i.d., αPD-1 i.v., Melan-A VLP (◆, purple, n = 5). ** denotes statistical by unpaired, two tailed Student's t test ($p < 0.005$). Error bars indicate SEM.

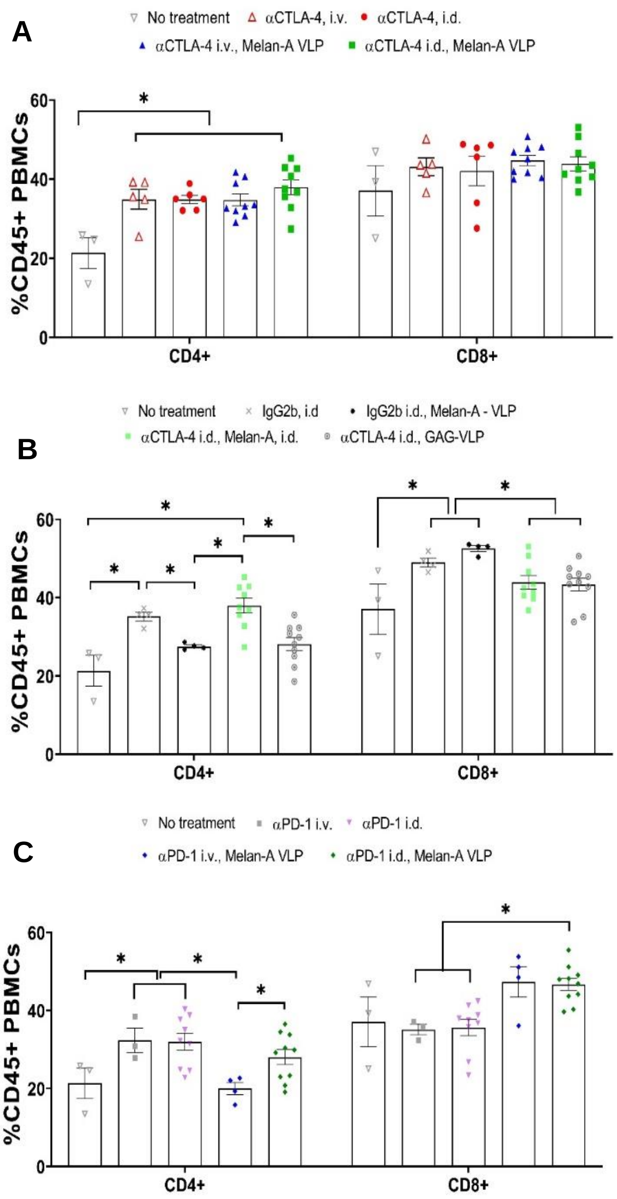

Figure S6. %CD4^{+} and CD8^{+} cells in PBMCs. (**A**). Delivery of αCTLA-4 monotherapy and in combination with Melan-A VLP increases the %CD4^{+} T cells in PBMCs. No treatment (∇, n = 3), αCTLA-4 administered intravenously (i.v.) (△, red, n = 5), αCTLA-4 administered intradermally (i.d.) (●, red, n = 6), αCTLA-4 i.v., Melan-A VLP i.d (▲, blue, n = 9), αCTLA-4 i.d., Melan-A VLP i.d. (■, green n = 9). (**B**) Melan-A VLP and Gag-VLP change the distribution of CD4^{+} and CD8^{+} T cells in PBMCs. No treatment (∇, n = 3), IgG2b i.d. (X, n = 4), IgG2b i.d., Melan-A VLP (✱, n = 4), αCTLA-4 i.d., Melan-A VLP i.d. (■, green, n = 9), αCTLA-4 i.d., Gag-VLP i.d. (○, n = 9). (**C**) Lymphatic delivery of αPD-1 in combination with Melan-A VLP changes the distribution of CD4^{+} and CD8^{+} T cells in PBMCs. No- treatment (∇, n = 3), αPD-1 i.v (■, grey, n = 3), αPD-1 i.d (▼, pink, n = 9), αPD-1 i.v., Melan-A VLP i.d (◆, blue, n = 4), αPD-1 i.d., Melan-A VLP i.d. (◆, green, n = 10). * denotes statistical significance using unpaired, two-tailed Student's t test ($p < 0.05$). Error bars indicate SEM.
